# Octahedral ruthenium (II) polypyridyl complexes as antimicrobial agents against mycobacterium

**DOI:** 10.7717/peerj.3252

**Published:** 2017-04-27

**Authors:** Guojian Liao, Zhengyuan Ye, Yunlu Liu, Bin Fu, Chen Fu

**Affiliations:** College of Pharmaceutical Sciences, Southwest University, Chongqing, China

**Keywords:** Ruthenium(II) complexes, Reactive oxygen species, Antibacterial activity

## Abstract

Tuberculosis is one of the world’s deadliest infectious disease with 1.5 millions deaths annually. It is imperative to discover novel compounds with potent activity against *M. tuberculosis*. In this study, susceptibilities of *M. smegmatis* to the octahedral ruthenium(II) polypyridyl complexes, **1** {[(bpy)_3_Ru] (PF_6_)_2_ (bpy = 2,2′-bipyridine)}, **2** {[(phen)_2_Ru(dppz)](PF_6_)_2_ (phen = 1,10-phenanthroline, dppz = dipyridophenazine)} and **3** {[(phen)_3_Ru](PF_6_)_2_} were measured by broth microdilution and reported as the MIC values. Toxicities of complex **3** to LO2 and hepG2 cell lines were also measured. Complex **2** inhibited the growth of *M. smegmatis* with MIC value of 2 µg/mL, while complex **3** was bactericidal with MIC value of 26 µg/mL. Furthermore, the bactericidal activity of complex **3** was dependent on reactive oxygen species production. Complex **3** showed no cytotoxicity against LO2 and hepG2 cell lines at concentration as high as 64 µg/mL, paving the way for further optimization and development as a novel antibacterial agent for the treatment of *M. tuberculosis* infection.

## Introduction

Tuberculosis (TB) affects 9.6 million people each year and leads to 1.5 millions deaths annually, which is exacerbated by the emergence of multidrug resistance (Engstrom, 2016; Glaziou et al., 2014). WHO estimates that there are approximately 48,000 cases of multidrug resistant TB (MDR-TB ) which are resistant to first-line treatment drugs, with around 10% of these being extensively drug-resistant tuberculosis (XDR-TB) that do not respond to some of the second-line drugs (Glaziou et al., 2014; Matteelli, Roggi & Carvalho, 2014). Hence, it is extremely desired to develop novel classes of antibacterial compounds with new mode of action to avoid existing resistance to established targets.

Due to the success of *cis*-platinum anticancer agents, there has been considerable interest in the development of therapeutic agents based upon other transition metals and ruthenium(II) complexes in particular, because of their well known interaction with nucleic acids, which are generally believed to be a target for many metal-based drugs (Abid, Shamsi & Azam, 2016; Madureira et al., 2013). Octahedral ruthenium complexes have shown tremendous applications in different areas of biological research (Kostova, 2006; Li, Collins & Keene, 2015). Compared to small organic molecules, octahedral ruthenium complexes possess two advantages as DNA binding agents (Erkkila, Odom & Barton, 1999; Song & Yang, 2001). Firstly, the coordination complexes offer a unique modular system through the centered metal acts as a structural center, holding a rigid, three-dimensional scaffold of ligands. The DNA-binding and recognition properties thereby could be easily varied by replacing the ligands. Secondly, ruthenium complexes bear rich photophysical and electrochemical properties, which have led to more extensive fields, such as fluorescent markers and electrochemical probes.

**Figure 1:**
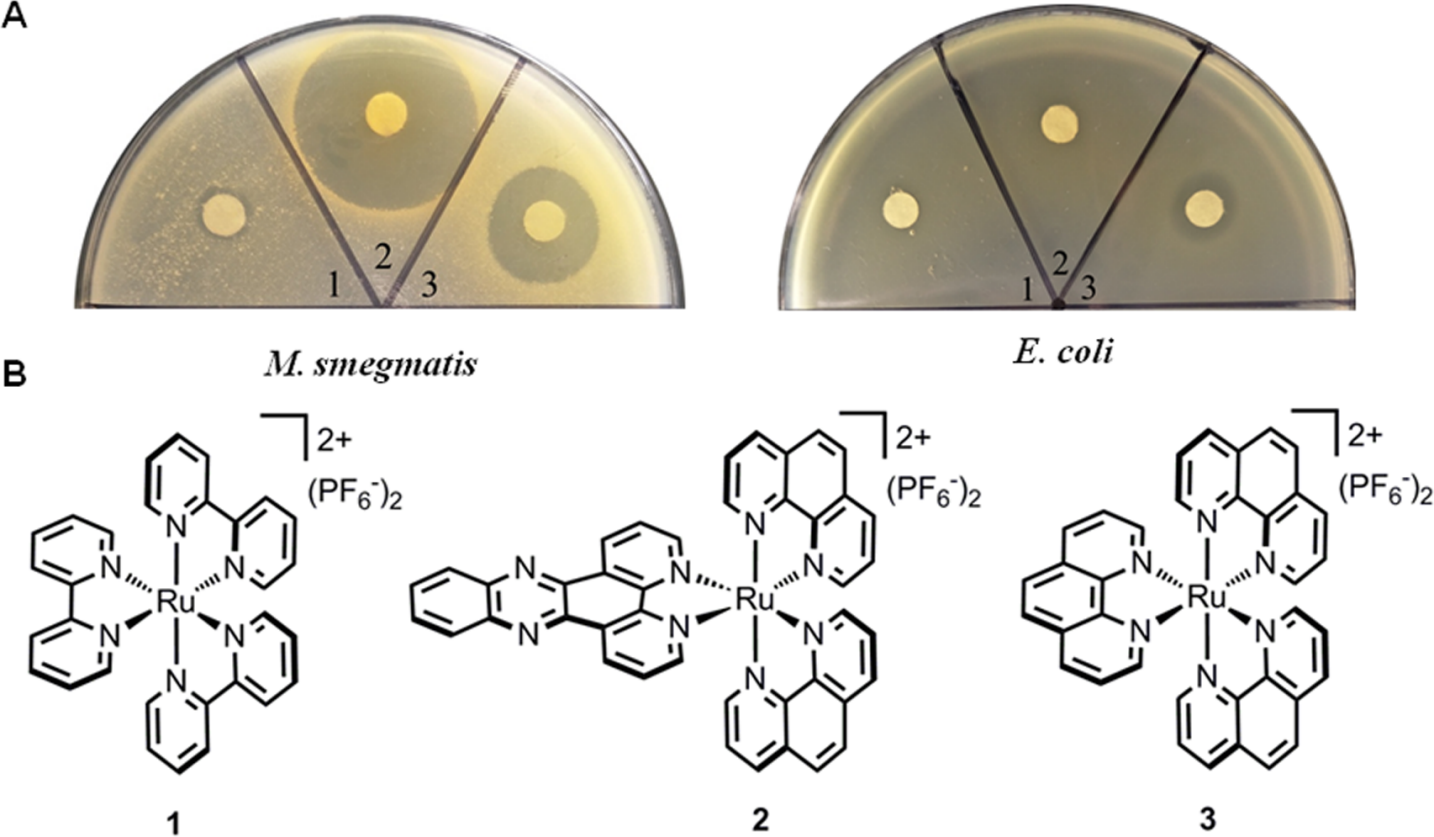
Antimicrobial activities of ruthenium complexes. (A) Strains including *E. coli* ATCC25922 and *M. smegmatis* mc^2^155 were used as indicator strains; (B) Chemical structures of complexes (**1**–**3)**.

In order to gain novel antibacterial agents for the treatment of *M. tuberculosis* infection, we investigated the antibacterial activities of the representative ruthenium polypyridyl complexes, **1**, **2** and **3** (Fig. 1), and found that they displayed potent antimicrobial activity against *M. smegmatis*, a nonpathogenic strain often used as a surrogate for the detection of compounds which are inhibitory to the growth of *M. tuberculosis* (Chaturvedi et al., 2007; Kostova, 2006). The bactericidal effect of complex **3** on *M. smegmatis* was dependent on ROS production. The dramatic killing of *M. smegmatis* by ruthenium complexes argues for the syntheses of series of derivatives to further investigate their effect on *M. tuberculosis*.

## Materials and Methods

### Tested compounds

Ruthenium complexes **1** and **3** (Ye et al., 2013), **2** (Liu et al., 2016), were prepared exactly according to the published procedures. ^1^H-NMR spectra were recorded on a Bruker Advance (400 MHz) at ambient temperature. The NMR data of **1**, **2** and **3** are listed as follows, which are consistent with the reported literatures respectively.

**1**: ^1^H-NMR (CD_3_CN): *δ* (ppm) 8.53 (d, *J* = 8.1 Hz, 6H), 8.08 (td, *J* = 8.0, 1.4 Hz, 6H), 7.76 (d, *J* = 5.6 Hz, 6H), 7.42 (ddd, *J* = 7.4, 5.6, 1.0 Hz, 6H);

**2**: ^1^H-NMR (d^6^-DMSO): *δ* (ppm) 9.62 (d, *J* = 8.1 Hz, 2H), 8.80 (t, *J* = 8.0 Hz, 4H), 8.54 (dd, *J* = 6.5, 3.5 Hz, 2H), 8.40 (s, 4H), 8.28 (d, *J* = 5.0 Hz, 2H), 8.25–8.15 (m, 4H), 8.05 (d, *J* = 5.0 Hz, 2H), 7.90 (dd, *J* = 8.0, 5.6 Hz, 2H), 7.78 (ddd, *J* = 15.2, 8.0, 5.0 Hz, 4H);

**3**: ^1^H-NMR (CD_3_CN): *δ* (ppm) 8.59 (dd, *J* = 8.3 Hz, *J* = 1.1 Hz, 6H), 8.25 (s, 6H), 8.01 (dd, *J* = 5.3 Hz, *J* = 1.1 Hz, 6H), 7.62 (dd, *J* = 8.2 Hz, *J* = 5.2 Hz, 6H).

Complex **1**-**3** were dissolved in 100% dimethyl sulfoxide (DMSO) and stored as frozen stocks at a concentration of 10 mM or 10 mg/mL. 2,2′-bipyridyl, thiourea and norfloxacin were bought from Sangon Biotech Co., and their stock solutions were freshly prepared, filter-sterilized, and used at indicated concentrations.

### Bacterial and fungal strains and media

Two *S. aureus* isolates, a methicillin-susceptible *S. aureus* (MSSA) strain (ATCC25923) and methicillin-resistant MRSA strain (ATCC33591), *M. smegmatis* mc^2^ 155 and two Gram-negative isolates, *Escherichia coli* ATCC25922, *Pseudomonas aeruginosa* PAO1, and fungal pathogens *Cryptococcus neoformans* H99, *Candida albicans* ATCC90028 were used for all tests. Disc diffusion assays were performed according to CLSI guidelines. Bacteria were grown in TSB medium; *C. neoformans* and *C. albicans* were grown in Yeast Extract Peptone Dextrose (YPD) medium; *M. smegmatis* mc^2^ 155 was grown in 7H9 liquid medium (Difco) supplemented with 0.05% w/v Tween 80, 0.5% glycerol and 0.5% glucose or were grown on 7H10 agar supplemented with 1% glycerol and 0.5% glucose. These strains were stored as glycerin stock in −80 °C.

### Minimum inhibitory concentration (MIC) susceptibility assay

MIC for complex **3** was determined by the broth micro-dilution method as recommended by Clinical and Laboratory Standards Institute (CLSI) (Zhai et al., 2012). In a 96-well plate, ten two-fold serial dilutions of each compound was made in a final volume of 100 µL TSB for bacteria, RPMI1640 for fungi or 7H9 for *M. smegmatis*. Each well was inoculated with 10^5^ bacterial cells or 10^2^ fungal cells at the initial time of incubation, prepared from a fresh log phase culture. Bacteria were incubated at 37 °C for 24 h and fungi were cultured at 37 °C for 48 h. The MIC was defined as the lowest concentration of antibiotic with no visible growth. The minimum bactericidal concentrations (MBCs) were defined as those at which at least 99% of cells were killed compared to the original inocula. DMSO served as the vehicle and negative control in each microdilution MIC assay.

### ROS determination

The Reactive Oxygen Species Assay Kit (Beyotime Institute of Biotechnology, Haimen, Jiangsu, China) based on 2′,7′-dichlorodihydrofluorescein diacetate (DCFH-DA) was utilized and the ROS production was measured as performing the manufacturer’s instructions (Li et al., 2015). Briefly, exponential-phase cultures were washed twice with 1×PBS and incubated with 10 mM DCFH-DA in 37 °C for 20 min. They were then washed twice with 1×PBS and re-suspended in PBS, before treating them with complex **3**. Aliquots were taken and the fluorescence intensity was monitored, using a Tecan Infinite 200 PRO microplate reader with excitation at 488 nm and emission at 525 nm. Relative ROS production was expressed as a percentage of DCF fluorescence of the untreated control.

### Effect of iron chelator and hydroxyl radical scavenger on antimicrobial activity

Subinhibitory concentrations of 2,2′-bipyridyl (250 µM; 50% MIC) and thiourea (100 mM; 50% MIC) were added to bacterial cultures 10 min prior to initiation of antimicrobial treatment. The cultures were then processed as the antimicrobial susceptibility assays described above (Duan et al., 2016).

### MTT assay

The hepatocyte cell line LO2 (Nanjing KeyGen Biotech, Nanjing, China), originating histologically from normal human liver tissue immortalized by stable transfection with the hTERT gene and hepatocellular Carcinoma Cell hepG2 were used for the cytotoxicity assessment (Huang et al., 2003; Zhang et al., 2009). The cells were grown in RPMI medium with 10% fetal bovine serum, penicillin, and streptomycin at 37 °C with 5% CO_2_. Cells (5 ×  10^3^/well) were added to 96-well plates and incubated with serial dilutions of compounds for 24 h. Subsequently, cell viability was quantified by addition of MTT, and plates were incubated for an additional 4 h at 5% CO_2_. Absorbance readings obtained at an A_490_nm (OD_490_) were used to calculate viability referenced against cells grown with no test compound.

## Results and Discussion

### Antimicrobial activities of the ruthenium complexes

The activities of ruthenium complexes **(1–3)** against *E. coli* and *M. smegmatis* were tested, and the results were shown in Fig. 1. Both complex **2** and **3** displayed impressive activities against *M. smegmatis.* Additionally, complex **3** exhibited little influence on the growth of *E. coli*. While *M. smegmatis* was highly susceptible to be killed by complexes **2** and **3**, the MIC and MBC were determined. Complexes **2** and **3** prevented the growth of *M. smegmatis* with MIC values of 2 and 26 µg/mL, respectively. The MBC was 10 µg/mL for complex **2** and 26 µg/mL for complex **3**. These results indicated that complex **2** may be bacteristatic (MBC/MIC = 5), while complex **3** may be bactericidal (MBC/MIC = 1). Complex **2** showed equal or better activity to first-line anti-tuberculosis drugs, such as norfloxacin, rifampicin and isoniazid, which had MIC values of 2 µg/mL, 1 µg/mL and 8 µg/mL, respectively. In addition to their potency, the activities of complex **2** and **3** were highly species-selective (Table 1). The complexes displayed no activity against any Gram-positive, Gram-negative bacteria or fungal species tested (>64 µg/mL), indicating that their target may be unique to mycobacteria.

**Figure 2:**
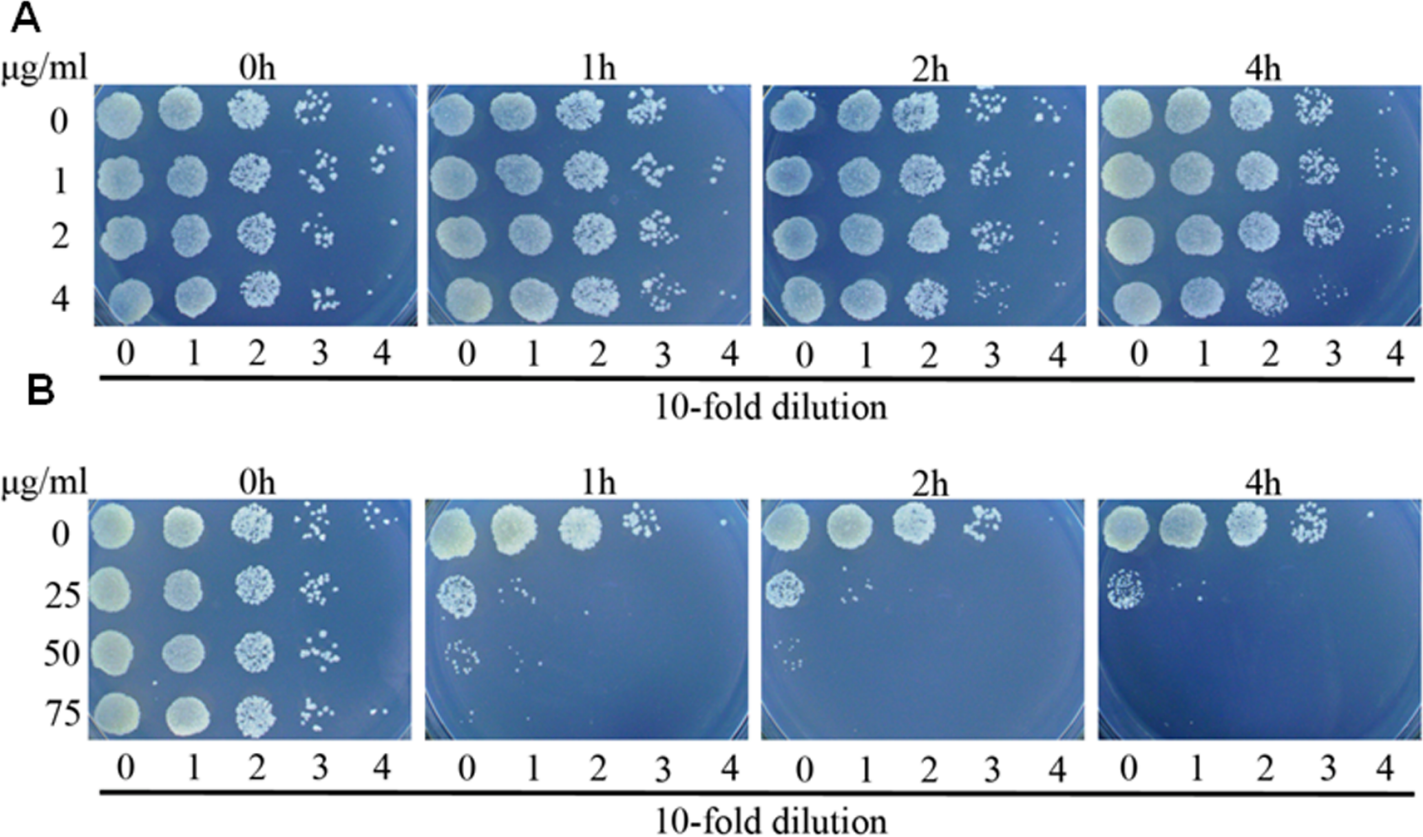
Complex 2 (A) was bacteriostatic and complex 3 (B) was bactericidal against *M. smegmatis*. Bacterial cells were inoculated 7H9 medium, and cultured without any drug or in the presence of complex **2** or **3** at various concentrations. At the indicated time points, aliquots of cell suspension were transferred and plated on drug-free 7H9 medium after 24 more hours of incubation.

### Antibacterial mechanism of complex 2 and 3

To further explore the activities of complex **2** and **3** against *M. smegmatis*, we performed time killing experiments. *M. smegmatis* was exposed to concentrations ranging from 0.5 fold to 2 folds MIC values of complex **2** or complex **3**. Survival was measured after 1, 2 and 4 h exposure at 37 °C . As displayed in Fig. 2, it was found that complex **2** just inhibited the growth of strain even at the concentration of 2 folds MIC, demonstrating that complex **2** was a bacteriostatic agent. In contrast to complex **2**, complex **3** exhibited potent bactericidal activity at the concentration of 1 fold MIC, decreasing bacterial numbers by over 2 logs with 1 h of treatment.

**Table 1:** Activity of complexes **1**–**3** against pathogenic microorganisms.

Organisms	Complex 1	Complex 2	Complex 3	MIC (µg/mL)	Rifampicin	Isoniazid
				norfloxacin		
*M. smegmatis*	>64	2	26	2	1	8
*S. aureus* (MSSA)	>64	>64	>64	ND	ND	ND
*S. aureus* (MRSA)	>64	>64	>64	ND	ND	ND
*P. aeruginosa*	>64	>64	>64	ND	ND	ND
*E. coli*	>64	>64	>64	ND	ND	ND
*C. albicans*	>64	>64	>64	ND	ND	ND
*C. neoformans*	>64	>64	>64	ND	ND	ND

**Notes.**

The MIC was determined by broth microdilution assay.

MSSA methicillin-sensitive *S. aureus* MRSA methicillin-resistant *S. aureus* ND non-determined

**Figure 3:**
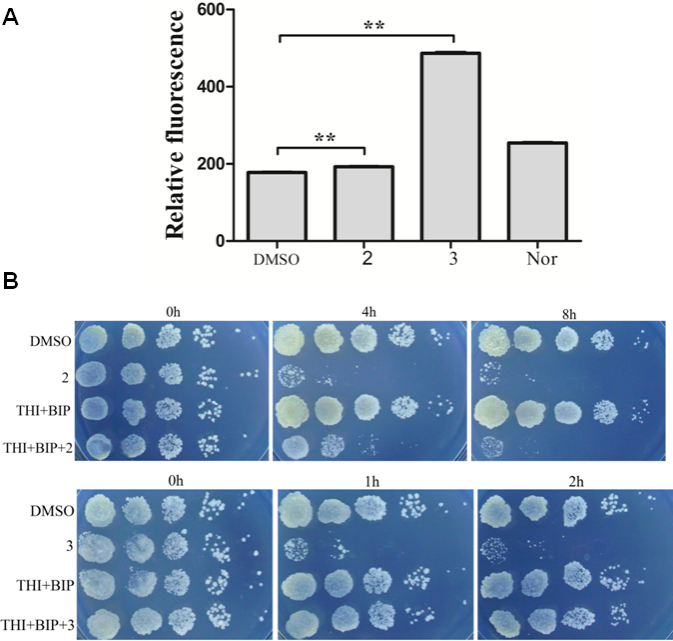
Complex 3 triggered endogenous ROS production in *M. smegmatis*. (A) Percentages of intracellular increase in ROS generation in the presence of 10 folds of MIC of complex 2 and **3**. Norfloxacin represents the positive control for ROS production. Data are shown as mean ± SD of triplicate wells. ^∗^*P* < 0.05; ^∗∗^*P* < 0.01; (B) Effects of a ferrous chelator and a hydroxyl radical scavenger on complex **2** and **3** lethality. Exponentially growing *M. smegmatis* cells were preincubated with 250 µM biphyridyl and 100 mM thioura for 10 min before they were treated with 10 folds of MIC of complex **2** and **3** for 4 or 8 h. Three replicate experiments were performed, and each had similar results.

ROS production is observed with antibiotics from a variety of bactericidal antibiotic families and ROS production has been proposed to be key factors in antimicrobial lethality (Wang et al., 2010). We next examined the possibility that lethality of complexes **2** and **3** was associated with ROS production. A qualitative assessment was carried out to determine intracellular ROS formation after incubation of *M. smegmatis* with complex **2** and **3**. Figure 3A presented fluorescent intensities of the amount of ROS production in the bacteria. The results showed that very low levels of ROS production in the control bacteria (DMSO). However, bacteria treated with complex **2** and **3** exhibited greater fluorescent intensities, indicating higher amounts of ROS formation in the bacteria with complex **2** and **3** treatment (*P* < 0.01). Particularly, the fact that complex **3** treatment exhibited a larger generation of ROS than complex **2** and norfloxin indicated that ROS formation may be the key factor of lethality of complex **3**.

**Figure 4:**
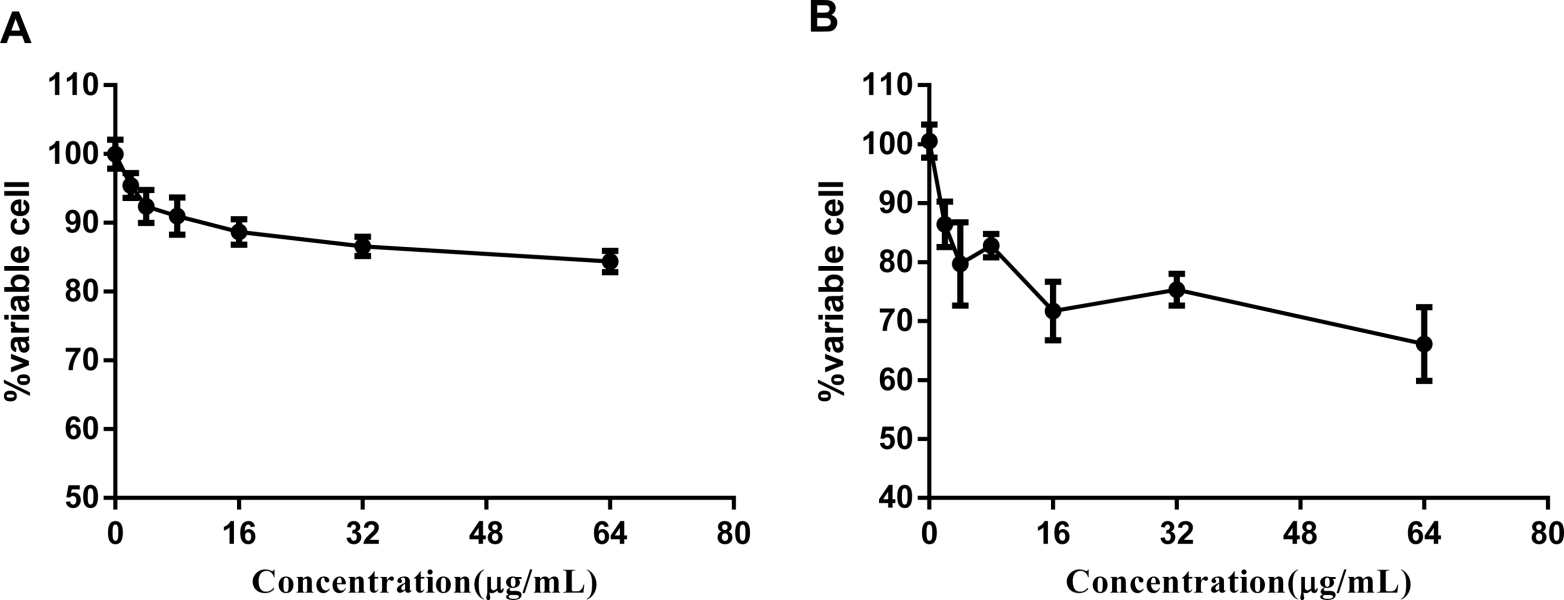
Complex 3 displayed modest cytotoxicity against LO2 (A) and hepG2(B) cell line.

To further investigate the contribution of ROS formation to the lethality of complex **2** and **3**, exponentially growth cells were treated with biphyridyl (an iron chelator) that interferes with the Fenton reaction and thiourea (a hydroxyl radical scavenger). Both thiourea and biphridyl could protect bacteria from the lethality of complex **3**, but not from that of complex **2**. The co-treatments reduced complex **3**-mediated killing by 10 to 100 folds, but had marginal activity on complex **2** (Fig. 3B). It was in line with the observation that complex **3** could trigger a large amount of ROS production. Taken together, these results suggested that the bactericidal activity of complex **3** might be the result of accumulated hydroxyl radicals.

### Cytotoxicity of complex 3

There are a wide variety of related ruthenium (II) complexes that exhibit high cytotoxicity against eukaryotic cell lines (Liu, Ji & Mei, 2004). We used immortal hepatic cell line LO2 and human liver cancer cell hepG2 to determine the cytotoxicity of ruthenium complex **3**. The results indicated that complex **3** displayed modest cytotoxicity against both LO2 and hepG2 cell lines with IC_50_ values of greater than 64 µg/mL (Fig. 4).

## Conclusion

In summary, we identified two typical octahedral ruthenium complexes that could selectively inhibit the growth of *M. smegmatis.* The results showed that they possessed different antimicrobial mechanisms: complex **2** could significantly inhibit the growth of *M. smegmatis* with MIC value of 2 µg/mL, while complex **3** was proposed to be bactericidal with MIC value of 26 µg/mL and its bactericidal activity was dependent on reactive oxygen species production. Moreover, both complexes showed no cytotoxicity for hepatic cell line LO2 and human liver cancer cell hepG2 cell line at the concentration as high as 64 µg/mL. To the best of our knowledge, no previous study has reported that these octahedral ruthenium polypyridyl complexes being utilized as the potential antimicrobial agents for *M. smegamtis*. Based on the results, we are convinced that these ruthenium complexes can serve as a useful scaffold for further modification to develop novel antibacterial agents for the treatment of *M. tuberculosis* infection.

##  Supplemental Information

10.7717/peerj.3252/supp-1 Supplemental Information 1 MTT assay of complex 2 and complex 3

10.7717/peerj.3252/supp-2 Supplemental Information 2 MIC value of complex 2 and 3

## Additional Information and Declarations

### Data Availability

The following information was supplied regarding data availability: The raw data has been supplied as a Supplementary File.
